# Deviant Peer Affiliation, Depression, and Adolescent Non-Suicidal Self-Injury: The Moderating Effect of the OXTR Gene rs53576 Polymorphism

**DOI:** 10.3390/children11121445

**Published:** 2024-11-27

**Authors:** Jingjing Li, Chengfu Yu

**Affiliations:** 1 School of Health Management, Guangzhou Medical University, Guangzhou 511436, China; 2023990019@gzhmu.edu.cn; 2 Department of Psychology, Research Center of Adolescent Psychology and Behavior, School of Education, Guangzhou University, Guangzhou 510006, China

**Keywords:** adolescent, deviant peer affiliation, depression, non-suicidal self-injury (NSSI), OXTR gene rs53576 polymorphism

## Abstract

Background: Adolescent non-suicidal self-injury (NSSI) has emerged as a progressively widespread and significant public health concern on a global scale. Research has increasingly documented a positive linkage between deviant peer affiliation and adolescent NSSI; however, there is little known about the underlying moderating or mediating mechanism of NSSI. According to the gene × environment interaction perspective, the current study investigated the intermediary function of depression in linking deviant peer affiliation to NSSI among adolescents, while also considering the moderating effect of the OXTR gene rs53576 polymorphism on this intermediary process. Methods: A total of 469 adolescents (Mean_age_ = 12.81 years; SD = 0.47 years) anonymously finished the study questionnaires. This study used structural equation modeling analysis to verify a moderated mediation model. Gender, age, and family financial difficulties were used as covariates. Results: Mediation analyses suggested that the positive connection between deviant peer affiliation and adolescent NSSI was mediated by depression. Moreover, the moderated mediation analyses revealed that deviant peer affiliation increased depression levels, which in turn contributed to increased NSSI among adolescents with the AA genotype. Nevertheless, the correlation failed to reach statistical significance among adolescents possessing the GA and GG genotypes. Conclusions: These findings emphasize depression’s potential as a bridge linking deviant peer affiliation to adolescent NSSI. The AA genotype of the OXTR gene rs53576 emerges as a critical risk factor in the enhancement of this indirect effect. This study provides valuable perspectives for designing intervention strategies aimed at reducing adolescent NSSI.

## 1. Introduction

Adolescence is a period of rapid physical and mental development for an individual. It is also a special period when an individual’s psychology and behavior transition from immaturity to maturity. During this period, adolescents often face pressure from family, peers, teachers, and studies, so they are inclined to exhibit a higher incidence of psychological and behavioral problems. Non-suicidal self-injury (NSSI) is a psychopathological behavior that occurs frequently in adolescents and refers to the intentional and repeated injury of an individual’s own body, including cutting, burning, and stabbing [1]. Studies conducted recently suggest that the prevalence of NSSI among adolescents spans from 15% to 25% [2,3,4]. Many empirical studies indicate that adolescent NSSI is a robust risk predictor of suicide attempts and suicidal ideation [4]. Therefore, ascertaining the risk factors and the underlying causes behind adolescents’ susceptibility to NSSI is imperative.

### 1.1. Deviant Peer Affiliation and NSSI

Deviant peer affiliation is perceived as a key determinant of adolescent NSSI [5,6]. From the viewpoint of the observation learning theory of NSSI, adolescents can learn this risky behavior from their friends by observing it [1]. The General Strain Theory [7] plays a crucial role in understanding how NSSI is linked to deviant peer affiliation. According to GST [7], deviant affiliation is an important source of pressure in adolescents. Adolescents may be too inexperienced or otherwise unable to remove the presence of trouble from their lives. This sense of helplessness can create a feeling of oppression. To cope with these troubling feelings, individuals may resort to pathological behaviors or respond in a non-adaptive manner [7,8,9]. Being affiliated with deviant peers can readily serve as a trigger for stress in life, which, in turn, can lead to long-lasting negative effects on adolescents [5,10]. These long-lasting negative effects caused by deviant peers result in challenges in school or in social adaptability and increase the possibility of involvement in risky behaviors, such as NSSI [6,11]. Deviant peer affiliation can simultaneously increase the possibility of thwarted belongingness and the feeling of entrapment; therefore, adolescence may produce a sense of isolation and alienation from social support, subsequently increasing NSSI. Empirical research has also shown that deviant peer affiliation is indicative of a higher predisposition for adolescent NSSI [6,11]. For instance, Syed et al. (2020) [6] found that NSSI among peers could be contagious, and teenagers who reach out to their peers’ NSSI are more likely to go through NSSI themselves. Similarly, Hilt and Hamm (2014) [12] reported that NSSI can be learned through peer observation. The significance of deviant peer affiliation in elevating the incidence of NSSI among adolescents is underscored by these findings.

### 1.2. Mediating Effect of Depression

A large body of studies underscored the role of depression as a significant conduit between environmental stressors and adolescent self-harming tendencies [13,14,15]. There is some indirect evidence to support the intermediary role of depression between adolescent NSSI and deviant peer affiliation. First, deviant peer affiliation was found to be a strong predictor of depression in a range of empirical research [14,16,17]. For example, Cheng and Li (2017) [18] revealed that affiliation with deviant peers escalates the chance of adolescents falling into depression. Similarly, Cole et al. (2014) [19] reported that teenagers who are affiliated with deviant peers are more susceptible to experiencing low self-efficacy in pursuing goals and negative self-perception, which, in turn, increased the vulnerability to depression.

Second, adolescents with depression are more likely to be involved in NSSI [20,21,22]. Based on the affect regulatory model of self-injury [23], when adolescents experience depression, they often adopt NSSI as a coping mechanism because of their immature physical and mental development and poor emotional management ability. Some longitudinal research has shown that adolescent depression predicts subsequent NSSI. For example, Wu et al. (2019) [24] revealed that adolescent depression positively predicted NSSI after 6 months. Analogously, Prinstein et al. (2010) [11] established a statistically significant connection between depressive symptoms and the incidence of NSSI in adolescents, which was consistent across the 6-, 12-, and 18-month follow-up periods. Therefore, we put forward the following hypothesis:

**Hypothesis** **1.** *The influence of deviant peer affiliation on adolescent NSSI will be mediated by depression*.

### 1.3. Moderating Effect of OXTR rs53576

Although depression is generally regarded as a robust predictor of adolescent NSSI, not all adolescents were equally impacted by depression. In other words, there are individual differences in adolescent development; that is, adolescents with different characteristics (i.e., different genotypes) are affected differently by depression. According to the differential susceptibility model [19], teenagers with a certain genotype are not only more prone to psychological and behavioral problems caused by a negative environment but are also more likely to be influenced by a positive environment. In recent years, more researchers have concentrated on exploring the process by which NSSI occurs, focusing on the interplay between genetic and environmental factors. Taking into account previous findings, we suggest that the OXTR gene rs53576 polymorphism is an indispensable moderating variable in relation to the linkage between poor peer association, depression, and NSSI in adolescents. The OXTR gene rs53576 polymorphism includes three genotypes: GA, GG, and AA. Ample evidence from research has indicated that the OXTR gene rs53576 polymorphism is associated with a variety of internal and external adolescent problem behaviors [24,25,26]. Moreover, nowadays, an increasing body of research has revealed that compared to adolescents with the GA or GG genotype of the rs53576 polymorphism, teenagers with the AA genotype are more sensitive to the environment (i.e., deviant peers). Specifically, adolescents with the AA genotype showed more stress reactivity [20,27,28], depression [29], and emotion regulation difficulties [27] when exposed to a negative environment. On the other hand, adolescents with the AA genotype showed more positive adaptation when exposed to a positive environment. For example, Yu, Li, and Zhang (2021) [30] found that compared with GG and GA genotype adolescents, adolescents with the AA genotype were more susceptible to parent–child conflict. Specifically, adolescents possessing the AA genotype exhibited a strong positive correlation between parent–child conflict and NSSI, whereas adolescents with the GG or GA genotypes showed no discernible link between these two variables. In accordance with the empirical data and theoretical perspectives presented, we pose the following hypothesis:

**Hypothesis** **2.** *The OXTR gene rs53576 polymorphism will moderate the effects of deviant peer affiliation on adolescent depression and NSSI*.

The OXTR gene rs53576 polymorphism may operate as a modulator of how adolescent depression affects their NSSI. Wang et al. (2014) [31] found that the OXTR gene rs53576 polymorphism is significantly associated with individual “amygdala brain region function related to emotion regulation”. Although no empirical studies have directly investigated the regulatory function of the OXTR gene rs53576 polymorphism in the correlation between depression and NSSI, some findings provide indirect evidence. For example, people with the GA or GG genotype of OXTR rs53576 were prone to seek social support from others, especially when they faced negative emotion and showed decreased reactivity to negative events and depression [27,28,32]. Moreover, prior research has shown that rs53576 exerted a moderating influence on the connection between emotion and behavior. For example, Moons et al. (2014) [33] found that adolescents carrying the AA genotype displayed a heightened level of hostile behavior after experiencing stress-inducing emotions, in contrast to those with the GG and GA genotypes. Similarly, Choi et al. (2019) [34] revealed a significant interaction effect between maternal postpartum depression and the OXTR rs53576 genotype on eradicating problems in adolescents with the AA genotype, but the interaction effect was not significant in adolescents with the GA or GG genotype. Thus, it is our conjecture that the OXTR rs53576 gene polymorphism will exert an influence on the relationship linking adolescent NSSI to depression. Drawing upon the aforementioned theoretical frameworks and empirical data, we formulate the subsequent hypothesis:

**Hypothesis** **3.** *The OXTR gene rs53576 polymorphism moderates the relationship between depression and adolescent NSSI*.

**Figure 1:**
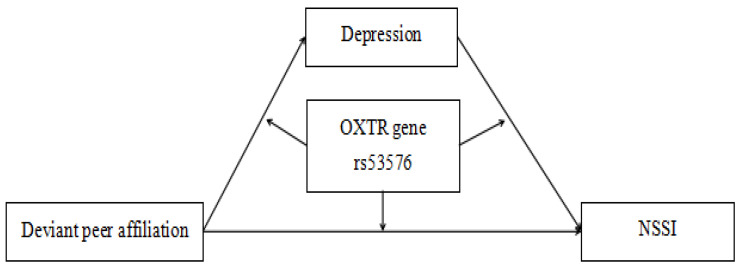
Proposed moderated mediation model.

In summary, the present study sought to determine whether depression mediates the connection between adolescent NSSI and deviant peer affiliation, as well as the moderating role of the rs53576 polymorphism. The suggested research model is presented in Figure 1.

## 2. Methods

### 2.1. Participants

A cohort of 469 junior school students, consisting of 246 males and 223 females aged 11 to 15 years (*Mean*_age_ = 12.81 years, *SD* = 0.47 years), was recruited from Guangdong Province, located in China. Overall, 92.1% of the participants resided in two-parent households. Additionally, a minority of the adolescents’ parents (specifically, 12.5% of mothers and 14.8% of fathers) possessed at least a bachelor’s degree.

### 2.2. Measures

#### 2.2.1. Deviant Peer Affiliation

A 12-item questionnaire invented by Zhu et al. (2015) [35] was employed to assess deviant peer affiliation. On a 5-point scale, with 1 denoting never and 5 signifying six or more occurrences, participants were asked to disclose how much deviant conduct their peers had engaged in over the last six months (e.g., “How many of your close friends were involved in fights in the past 6 months?”). A higher mean score corresponded to a stronger linkage to deviant friends with higher values. The study’s Cronbach’s α was 0.74.

#### 2.2.2. Depression

Adolescent depression was measured by the General Population Depression Scale [36]. The frequency of depression experienced by the participants in the last week was recorded through their self-reports. The scale included a total of 20 items (e.g., “I think my life is useless”). A 4-point score was used, with 1 indicating occasional or none and 4 indicating most of the time or duration. The average response across all questions was assessed, and a higher score corresponded to a more severe form of depression. The study’s Cronbach’s α was 0.74.

#### 2.2.3. NSSI

NSSI was assessed by the Chinese version of the NSSI questionnaire [25]. Participants were asked to rate the frequency of six NSSI behaviors in the past six months (e.g., “rubbing one’s skin”). A 6-point scale was used, in which 1 represents never and 6 represents several times a week. If the score is higher, then the NSSI is more severe. The study’s Cronbach’s α was 0.76. Also, 16% of participants said they had participated in NSSI within the previous six months.

#### 2.2.4. Genotyping

Saliva samples were utilized to extract genomic DNA, and the manufacturer’s instructions provided with the DNA Self Collection Kit were adhered to. Magnetic Bead Method Nucleic Acid Extraction Kit providing by the Wuhan Tianyi Huiyuan Biotechnology Co., Ltd., Wuhan, China. Subsequently, the rs53576 polymorphism was genotyped using the SNaPshot analysis system from Applied Biosystems (Waltham, MA, USA). The genotyping outcomes disclosed three distinct genotypes for OXTR rs53576: AA, GA, and GG.

#### 2.2.5. Family Financial Difficulties

A 4-item questionnaire designed by Wang, Li, and Zhang (2010) [31] was used to measure family financial difficulties. Participants were asked how frequently, during the previous six months, their family members had dealt with financial difficulty (e.g., “My family doesn’t have enough money to buy the food I like.”; “My family doesn’t have enough money to buy a good house.”). A 5-point score was used, with 1 indicating “never” and 5 indicating “always”. The study’s Cronbach’s α was 0.83.

### 2.3. Procedure

The study commenced following the approval from the institutional academic ethics review board and the procurement of consent from all participants, along with their parents. Initially, the participants were briefed on the study protocols and informed of their right to withdraw at any stage. Subsequently, guided by proficient psychology teachers and psychology majors pursuing graduate studies, the participants completed self-report scales within their classrooms. Upon finishing the questionnaires, they were requested to submit saliva samples. The entire process spanned 45 min.

### 2.4. Statistical Analysis

Given the low frequency of missing values, we replaced them with the mean of the observed values. Then, descriptive statistics and correlation analyses were performed. Finally, we analyzed the mediating and moderating effects using SPSS 26.0 PROCESS v3.4, employing the bootstrapping method with 5000 resamples.

## 3. Results

### 3.1. Descriptive Statistics

Table 1 displays the averages, standard deviations, and correlations. Both depression and NSSI are positively correlated with deviant peer affiliation. Depression was significantly correlated with NSSI.

### 3.2. Testing for the Mediation Effect of Depression

Table 2 exhibits the mediation model. After controlling for age, gender, and family financial difficulties, deviant peer affiliation significantly affected depression (*β* = 0.24, *SE* = 0.05, *t* = 5.34, *p* < 0.001, 95% CI [0.15, 0.33]), and depression positively affected NSSI (*β* = 0.29, *SE* = 0.05, *t* = 6.16, *p* < 0.001, 95% CI [0.19, 0.38]). Deviant peer affiliation had a noteworthy impact on NSSI as well (*β* = 0.10, *SE* = 0.05, *t* = 2.16, *p* < 0.05, 95% CI [0.01, 0.19]). The mediating impact of depression was found to be significant (indirect effect = 0.07, *SE* = 0.03, 95% CI [0.03, 0.13]).

### 3.3. Moderating Effect of the OXTR Gene rs53576 Polymorphism

**Figure 2:**
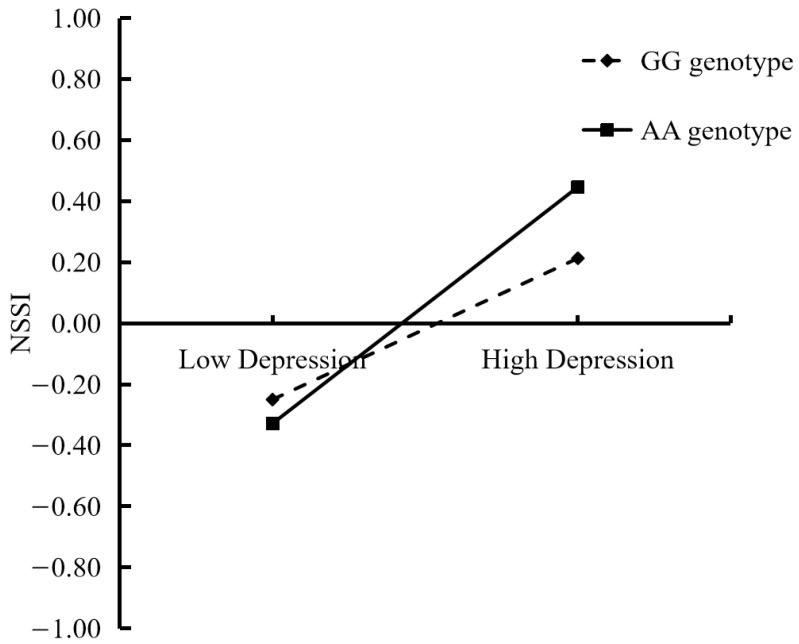
Interactive effect of depression and OXTR gene rs53576 polymorphism on adolescent NSSI.

Table 3 exhibits the moderated mediation model. After controlling for covariates (age, gender, and family financial difficulties), deviant peer affiliation had a significant positive connection with depression (*β* = 0.44, *SE* = 0.22, *t* = 2.12, *p* < 0.05, 95% CI [0.00, 0.89]). However, the main effects of genotype and the interaction effects of deviant peer affiliation and genotype were nonsignificant. Moreover, there was a significant interaction between depression and genotype AA (*β* = 0.28, *SE* = 0.15, *t* = 1.74, *p* < 0.05, 95% CI [0.02, 0.60]). According to the follow-up simple slope test, only adolescents with the AA genotype showed a positive and significant correlation between depression and NSSI (see Figure 2). Additionally, the main effects of deviant peer affiliation, depression, genotype AA and genotype GA; the interaction effects of deviant peer affiliation and genotype AA and genotype GA; and the interaction effects of depression and genotype GA on NSSI were nonsignificant.

We further investigated whether the OXTR gene rs53576 polymorphism conditioned the indirect connection, through depression, between deviant peer affiliation and adolescent NSSI. The indirect connection was only significant for adolescents with the AA genotype of the OXTR gene rs53576 polymorphism (conditional indirect effect = 0.08, *SE* = 0.04, 95% CI [0.02, 0.19]).

## 4. Discussion

There is strong empirical evidence that deviant peer affiliation affects the probability of adolescent NSSI. However, the essential mediating and moderating factors still constitute areas of inquiry that require further exploration. A moderated mediation model was developed and tested in this study. First, supporting hypothesis 1, we identified depression as a fundamental, underlying psychosocial mechanism that helps make clear why greater deviant peer affiliation is associated with more NSSI. When a person is faced with a deviant peer, the individual may produce corresponding psychological pressure, especially if the person is not equipped with good coping experience or ability. This kind of pressure can easily lead to depressive disorder, which, in turn, is associated with more NSSI [37]. The main reason why NSSI often occurs after experiencing deviant peer affiliation is that NSSI functions to manage emotions [16]. This finding is also in line with the individual–environment interaction model [38], which posits that environmental and individual factors are mutual and that how one functions has a significant impact on how the others function.

In line with hypothesis 3, we found that the OXTR gene rs53576 polymorphism moderated the connection between depression and adolescent NSSI, given that the moderating impact of the rs53576 polymorphism was mostly evident in the regulation of negative emotions (depression) caused by deviant peers. Specifically, there was no difference between GA and GG genotype carriers in the susceptibility to depression regarding adolescent NSSI, nor were both carriers susceptible to depression. Only AA genotype carriers were susceptible to depression. These findings suggest that the rs53576 polymorphism has an effect on adolescent NSSI in a recessive genetic way. The moderating effects of the rs53576 polymorphism were significantly different when different genetic types were used, which may be because the “correct” genetic type of the rs53576 polymorphism varies with the environment experienced by individuals [39]. The differential susceptibility model is supported by this finding [19]. The results also indicated that adolescents with GG and GA genotypes of the rs53576 polymorphism were not susceptible to depression and that their NSSI was not affected by depression. However, adolescents with the AA genotype were susceptible to depression: they are more likely to be negatively influenced by high levels of depression and show more NSSI, and more likely to be positively affected by low levels of depression and show less NSSI. Wang et al. (2014) [30] found that the amygdala function of AA genotype carriers as it relates to emotion regulation was lower, and AA genotype carriers had more difficulty in emotion management than those with GG or GA genotypes of the rs53576 polymorphism. This result helps to explain why adolescents with the AA genotype may show more NSSI when depressed: they cannot regulate their emotions when they face depression and other negative emotions and engage in NSSI to relieve these negative emotions.

The current study has several limitations. Firstly, we selected only middle school students as participants with strong homogeneity. Thus, it is necessary to confirm the ecological validity of this study. Secondly, the connection between NSSI and deviant peer affiliation was investigated using horizontal analysis in this cross-sectional study. However, the development of NSSI is a gradual process, and the impacts of deviant peer affiliation on adolescents are ongoing and extensive. Future research employing longitudinal designs would be invaluable in capturing the dynamic and long-term processes underlying the connection between deviant peer affiliation, depression, and NSSI. For example, Wu et al. (2021) [40] conducted a longitudinal study demonstrating how peer acceptance influences NSSI through depressive symptoms over time. NSSI is readily impacted by personal factors during adolescence. Last, the present study focused only on OXTR rs53576; the role of other genes should be explored in future studies. However, the present study provides crucial and preliminary data on the risk of deviant peer affiliation interactions for NSSI in youth. This study also supports a role of the OXTR gene rs53576 in moderating the effects of negative emotions, an attractive neurological finding that emphasizes the need for more research on this topic and other neurological systems in NSSI during adolescence. More importantly, looking at the results of this study, it is possible to find that adolescent NSSI has unique influencing factors and mechanisms. This reminds us to distinguish between various psychological issues, and the creation of targeted prevention and intervention plans will help reduce NSSI. In the future, when researchers formulate relevant prevention or intervention projects, they should take into account the differences in the cause of NSSI, formulate different prevention and intervention projects for NSSI, and maximize the use of resources for prevention and intervention. In summary, in the early and middle period of adolescence, there are distinctions between the NSSI mechanism and its impacting elements. The sensitivity of individuals is different due to their adaptations to the fields and environmental areas. During the process, these important aspects should be emphasized, and individual differences and a reasonable and efficient allocation of resources should be focused on. In addition, families, schools, and social institutions should pay attention to mental health education to solve the onset and progression of adolescent NSSI.

## Figures and Tables

**Table 1:** Descriptive statistics and correlations among variables.

	1	2	3	4	5	6	7	8
1. Gender	1.00							
2. Age	−0.04	1.00						
3. FFD	−0.03	0.05	1.00					
4. DPA	0.01	−0.03	0.21 ***	1.00				
5. Depression	0.08	0.08	0.18 ***	0.27 ***	1.00			
6. NSSI	0.05	−0.03	0.08	0.18 ***	0.31 ***	1.00		
7. Gene AA ^a^	0.09	0.05	0.01	0.02	−0.04	0.04	1.00	
8. Gene GA ^b^	−0.06	0.01	0.00	−0.02	0.02	−0.02	−0.85 ***	1.00
*Mean*	—	12.81	1.32	1.40	1.69	1.05	—	—
*SD*	—	0.47	0.55	0.48	0.43	0.20	—	—

Note: *** *p* < 0.001. The code for gender was 1 for male and 0 for female. Gene was dummy coded into two variables (^a^: AA = 1, GA = GG = 0; ^b^: GA = 1, AA = GG = 0). FFD = family financial difficulties, DPA = deviant peer affiliation, NSSI = non-suicidal self-injury, SD = standard deviation.

**Table 2:** Testing for the mediation effect of depression.

	Equation 1 (Depression)				Equation 2 (NSSI)			
	*β*	*SE*	*t*	95% CI	*β*	*SE*	*t*	95% CI
*Covariates*:								
Gender	0.17	0.09	1.91	[−0.01, 0.34]	0.42	0.09	0.47	[−0.13, 0.22]
Age	0.08	0.04	1.82	[−0.01, 0.17]	−0.05	0.04	−1.18	[−0.14, 0.03]
FFD	0.13	0.05	2.78 **	[0.04, 0.21]	0.01	0.05	0.21	[−0.08, 0.10]
*Study variables*:								
DPA	0.24	0.05	5.34 ***	[0.15, 0.33]	0.10	0.05	2.16 *	[0.01, 0.19]
Depression					0.29	0.05	6.16 ***	[0.19, 0.38]
*R* ^2^	0.10				0.11			
*F*	12.81 ***				11.44 ***			

Note: Values are standardized coefficients. * *p* < 0.05, ** *p* < 0.01, *** *p* < 0.001. The code for gender was 1 for male and 0 for female. FFD = family financial difficulties, DPA = deviant peer affiliation, NSSI = non-suicidal self-injury.

**Table 3:** Testing for the moderating role of the OXTR gene rs53576 polymorphism in the indirect relationship between deviant peer affiliation and NSSI via depression.

	Equation 1 (Depression)				Equation 2 (NSSI)			
	*β*	*SE*	*t*	95% CI	*β*	*SE*	*t*	95% CI
*Covariates*:								
Gender	0.18	0.09	2.01	[−0.00, 0.34]	0.02	0.10	0.19	[−0.19, 0.20]
Age	0.09	0.06	1.89	[−0.04, 0.21]	−0.06	0.05	−1.38	[−0.17, 0.02]
FFD	0.13	0.05	2.85 **	[0.03, 0.22]	0.00	0.04	0.02	[−0.08, 0.09]
*Study variables*:								
DPA	0.44	0.22	2.12 *	[0.00, 0.89]	0.01	0.08	0.04	[−0.12, 0.18]
Gene AA ^a^	−0.25	0.18	−1.51	[−0.63, 0.07]	0.24	0.11	1.43	[0.04, 0.47]
Gene GA ^b^	−0.16	0.18	−0.96	[−0.54, 0.17]	0.16	0.07	0.97	[0.02, 0.30]
DPA × Gene AA	−0.23	0.23	−1.04	[−0.68, 0.24]	0.10	0.12	0.44	[−0.13, 0.34]
DPA × Gene GA	−0.19	0.24	−0.84	[−066, 0.29]	0.08	0.10	0.34	[−0.12, 0.25]
Depression					0.10	0.06	0.67	[−0.01, 0.21]
Depression × Gene AA					0.28	0.15	1.74 *	[0.02, 0.60]
Depression × Gene GA					0.13	0.12	0.78	[−0.08, 0.40]
*R* ^2^	0.11				0.13			
*F*	6.88 ***				5.97 ***			

Note: Values are standardized coefficients. * *p* < 0.05, ** *p* < 0.01, *** *p* < 0.001. The code for gender was 1 for male and 0 for female. Gene was dummy coded into two variables (^a^: AA = 1, GA = GG = 0; ^b^: GA = 1, AA = GG = 0). FFD = family financial difficulties, DPA = deviant peer affiliation, NSSI = non-suicidal self-injury.

## Data Availability

The data are not publicly available due to privacy and anonymity considerations. The data presented in this study are available on request from the corresponding author.
